# Differential effects of 24-hydroxycholesterol and 27-hydroxycholesterol on β-amyloid precursor protein levels and processing in human neuroblastoma SH-SY5Y cells

**DOI:** 10.1186/1750-1326-4-1

**Published:** 2009-01-06

**Authors:** Jaya RP Prasanthi, Amber Huls, Sarah Thomasson, Alex Thompson, Eric Schommer, Othman Ghribi

**Affiliations:** 1 Department of Pharmacology, Physiology and Therapeutics, University of North Dakota School of Medicine and Health Sciences, Grand Forks, North Dakota 58202, USA

## Abstract

**Background:**

Activation of the liver × receptors (LXRs) by exogenous ligands stimulates the degradation of β-amyloid 1–42 (Aβ42), a peptide that plays a central role in the pathogenesis of Alzheimer's disease (AD). The oxidized cholesterol products (oxysterols), 24-hydroxycholesterol (24-OHC) and 27-hydroxycholesterol (27-OHC), are endogenous activators of LXRs. However, the mechanisms by which these oxysterols may modulate Aβ42 levels are not well known.

**Results:**

We determined the effect of 24-OHC and/or 27-OHC on Aβ generation in SH-SY5Y cells. We found that while 27-OHC increases levels of Aβ42, 24-OHC did not affect levels of this peptide. Increased Aβ42 levels with 27-OHC are associated with increased levels of β-amyloid precursor protein (APP) as well as β-secretase (BACE1), the enzyme that cleaves APP to yield Aβ. Unchanged Aβ42 levels with 24-OHC are associated with increased levels of sAPPα, suggesting that 24-OHC favors the processing of APP to the non-amyloidogenic pathway. Interestingly, 24-OHC, but not 27-OHC, increases levels of the ATP-binding cassette transporters, ABCA1 and ABCG1, which regulate cholesterol transport within and between cells.

**Conclusion:**

These results suggest that cholesterol metabolites are linked to Aβ42 production. 24-OHC may favor the non-amyloidogenic pathway and 27-OHC may enhance production of Aβ42 by upregulating APP and BACE1. Regulation of 24-OHC: 27-OHC ratio could be an important strategy in controlling Aβ42 levels in AD.

## Background

Cholesterol-enriched diets cause hypercholesterolemia and lead to increased levels of β-amyloid (Aβ) peptide in rabbit brain [1-3]. Because Aβ accumulation is a pathological hallmark of Alzheimer's disease (AD), high blood cholesterol levels may be a risk factor for AD in humans. However, because cholesterol homeostasis in the brain is regulated through *de novo *synthesis, with no or very poor transfer from the peripheral circulation due to the impermeability of the blood brain barrier (BBB) to plasma lipoproteins [4], the mechanisms by which cholesterol in the peripheral system increases Aβ levels in the brain are not fully understood.

In contrast to cholesterol, the side-chain oxidized oxysterols, 24-hydroxycholesterol (24-OHC) and 27-hydroxycholesterol (27-OHC), have the ability to cross lipophilic membranes into and out of the brain [5,6]. Increased cholesterol levels in plasma may result in increased levels of oxysterols and subsequent increased entrance of these compounds into the brain. Abnormal levels of these oxysterols in the brain might therefore be the trigger of increased Aβ production. Furthermore, oxysterols are endogenous activators of liver × activated receptors (LXRs), which have been shown to play a role in regulation of Aβ in the brain by mechanisms involving cholesterol transporters [7,8]. Although 24-OHC and 27-OHC have been demonstrated to modulate Aβ levels in primary cortical neurons [9], the mechanisms by which these oxysterols regulate Aβ production are not fully understood.

The aim of our study is to determine the extent to which and mechanisms by which 24-OHC and/or 27-OHC modulate Aβ generation in human neuroblastoma cells. Aβ is generated from β-amyloid precursor protein (APP) through an initial cleavage with the β-secretase, BACE1 [10-12]. Two forms of Aβ, a major species Aβ40 and a minor species Aβ42, are produced under physiological conditions. Cleavage of APP by α-secretase, on the other hand, leads to a non-amyloidogenic pathway [10]. We therefore determined the effect of the two oxysterols on levels of Aβ40, Aβ42, APP, secreted APP after α-secretase cleavage (sAPPα), and BACE1. We have also measured the effects of the oxysterols on levels of the ATP-binding cassette transporters ABCA1 and ABCG1, which regulate cholesterol transport and Aβ levels in cells.

## Results

### 27-OHC, but not 24-OHC, increases Aβ42 levels

**Figure 1:**
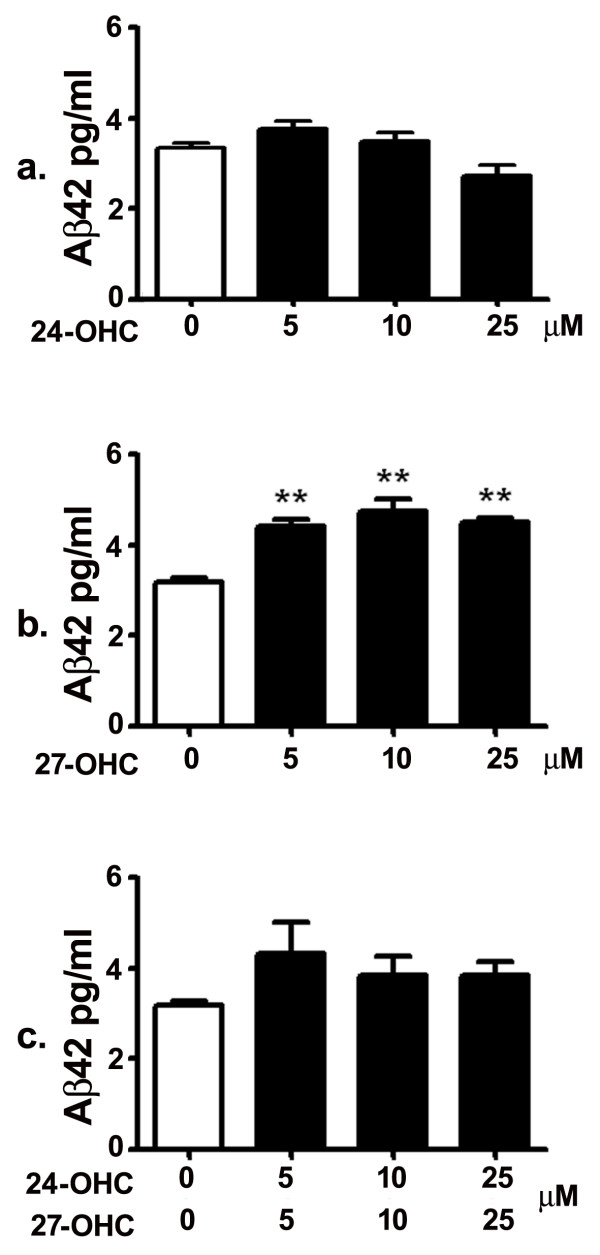
**27-OHC, but not 24-OHC, increases levels of secreted Aβ42**. While treatment with 5, 10 and 25 μM 24-OHC did not alter Aβ42 levels (a), treatment with 5, 10 and 25 μM 27-OHC significantly increased levels of Aβ42 compared to levels in medium of untreated cells (b). There was no difference in Aβ42 levels between untreated cells and cells treated with a mixture of 24-OHC + 27-OHC (c). **p < 0.01 (One way ANOVA followed by Dunnett's multiple comparison test).

Human neuroblastoma SH-SY5Y cells were treated with 5, 10 and 25 μM of 24-OHC, 27-OHC, or a mixture of 24-OHC and 27-OHC, and Aβ42 levels were determined with ELISA. ELISA measurements showed that treatment with 5, 10 or 25 μM 24-OHC did not induce significant changes in secreted Aβ42 levels compared to levels in medium of untreated cells (Fig. 1a). Conversely to 24-OHC, treatment with 5, 10 or 25 μM 27-OHC led to a substantial increase in Aβ42 levels (Fig. 1b). Treatment with a mixture of 24-OHC + 27-OHC did not induce significant changes in the levels of Aβ42 compared to levels from untreated cells or cells treated with 27-OHC (Fig. 1c). These results suggest that, although it doesn't reduce Aβ42 levels *per se*, 24-OHC, when added to 27-OHC, prevents the 27-OHC-induced significant increase in Aβ42 levels.

### 27-OHC increases levels of APP and BACE1

**Figure 2:**
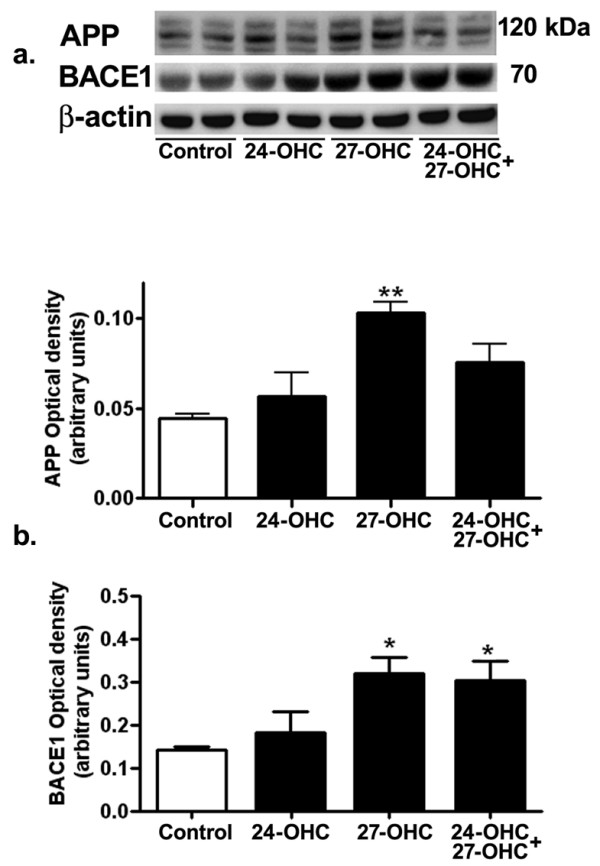
**27-OHC, but not 24-OHC, increases levels of APP and BACE1**. Representative Western blots (a) and densitometric (b) analysis demonstrating increased levels of APP with 27-OHC. No changes were found in levels of APP with 24-OHC or 24-OHC+ 27-OHC treatment. BACE1 levels were unchanged with 24-OHC treatment but significantly increased with 27-OHC or a mixture of 24-OHC + 27-OHC. *p < 0.05, **p < 0.01 (One way ANOVA followed by Dunnett's multiple comparison test).

In order to determine potential mechanisms involved in 27-OHC-induced increase in Aβ42 levels, we examined first the effects of 24-OHC and 27-OHC on levels of APP and BACE1. We chose to carry out our experiments with a concentration of 10 μM 24-OHC or 27-OHC. At 10 μM/mL concentration, 24-hydroxycholesterol has been shown to increase APP gene expression in human neuronal cells [13] and 27-hydroxycholesterol has been shown to inhibit neutral sphingomyelinase in human endothelial cells [14]. Western blot (Fig. 2a) and densitometric (Fig. 2b) analyses show the effect of 24-OHC, 27-OHC, or a mixture of 24-OHC + 27-OHC on levels of APP and BACE1. There was a significant increase in APP levels following treatment with 27-OHC. Treatment with 24-OHC or with a mixture of 24-OHC + 27-OHC did not statistically increase APP levels. Increase in APP levels with 27-OHC was associated with an increase in BACE1 levels. Treatment with 24-OHC did not significantly alter BACE1 levels. The mixture of 24-OHC + 27-OHC increased BACE1 levels significantly, indicating that 24-OHC does not reverse the effects of 27-OHC on BACE1 levels. These results suggest that 27-OHC-induced increase in levels of APP and BACE1 favors the amyloidogenic pathway that leads to increased Aβ42 production.

**Figure 3:**
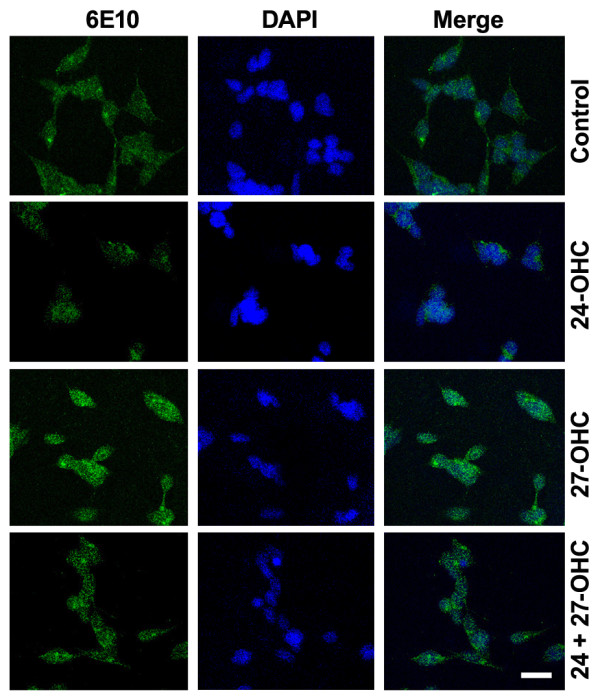
**Immunofluorescence staining of APP and Aβ increases with 27-OHC**. Immunostaining for APP and Aβ showed a decreased immunoreactivity to 6E10 antibody (green) in cells treated with 24-OHC compared treatment with 27-OHC. The immunoreactivity for 6E10 antibody in cells treated with a mixture of 24-OHC + 27-OHC is similar to that observed in control cells. DAPI (blue) was used as a nuclear counterstain. Bar 20 μm.

The immunofluorescence imaging (Fig. 3) showed a reduced immunoreactivity to 6E10, an antibody that detects full length APP as well as Aβ, in cells treated with 24-OHC in comparison to control cells. A substantial increase in 6E10 staining was observed in cells with 27-OHC compared to control cells or cells treated with 24-OHC. In cells treated with a mixture of 24-OHC+27-OHC, the intensity of the immunoreactivity to 6E10 antibody appears similar to that in control cells. These results are in accordance with the Western blot data showing increased APP and Aβ42 levels with 27-OHC compared to treatment with 24-OHC.

### 24-OHC increases processing of APP via the non-amyloidogenic pathway

**Figure 4:**
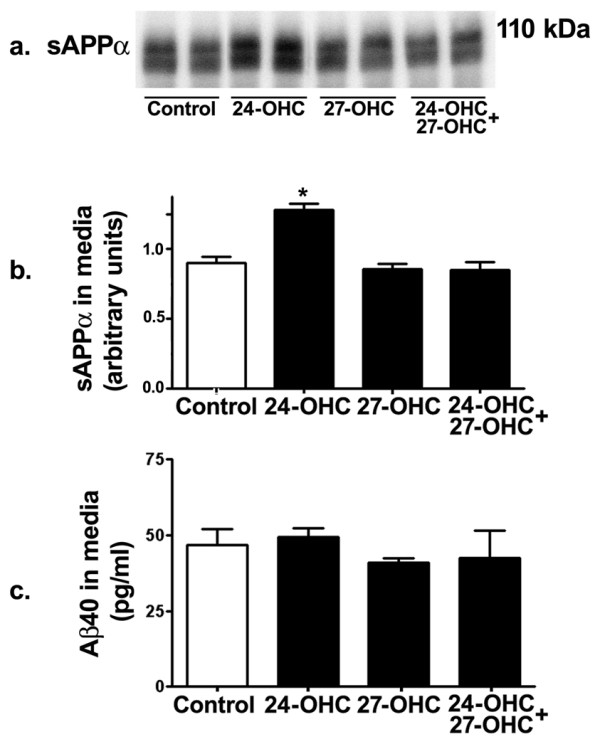
**24-OHC increases processing of APP via the non-amyloidogenic pathway**. Western blot (a) and densitometric analyses (b) demonstrating increased levels of sAPPα in medium of 24-OHC-treated cells. Treatment with 27-OHC or a mixture of 24-OHC + 27-OHC did not influence sAPPα levels. Levels of Aβ40 were not affected by treatment with 24-OHC, 27-OHC, or a mixture of 24-OHC + 27-OHC compared to levels in control cells (c). *p < 0.05 (One way ANOVA followed by Dunnett's multiple comparison test).

Extracellular sAPPα levels were determined with Western blot analysis in cell medium of control and of cells treated with 24-OHC, 27-OHC, and 24-OHC+27-OHC. Treatment with 24-OHC led to a substantial increase in sAPPα levels (Fig. 4a and 4b). No significant changes in sAPPα levels were observed in cells treated with 27-OHC or with a mixture of 24-OHC+27-OHC when compared to control cells (Fig. 4a and 4b). These results suggest that 24-OHC favors the processing of APP via the non-amyloidogenic pathway that precludes Aβ42 production.

To determine whether increased Aβ42 levels following treatment with 27-OHC is associated with reduced levels of Aβ40, the most abundant Aβ species in normal brain, Aβ40 levels in the medium were measured using ELISA. None of the 24-OHC, 27-OHC and 24-OHC + 27-OHC treatments altered Aβ40 levels (Fig. 4c). These results suggest that the increase in Aβ42 we found with 27-OHC originates from increased processing of APP by BACE1 and not from shift of APP processing from Aβ40 to Aβ42. Collectively, these results demonstrate that 24-OHC favors the non-amyloidogenic pathway while 27-OHC enhances the amyloidogenic pathway.

### 24-OHC, but not 27-OHC, increases ABCA1 and ABCG1 levels

**Figure 5:**
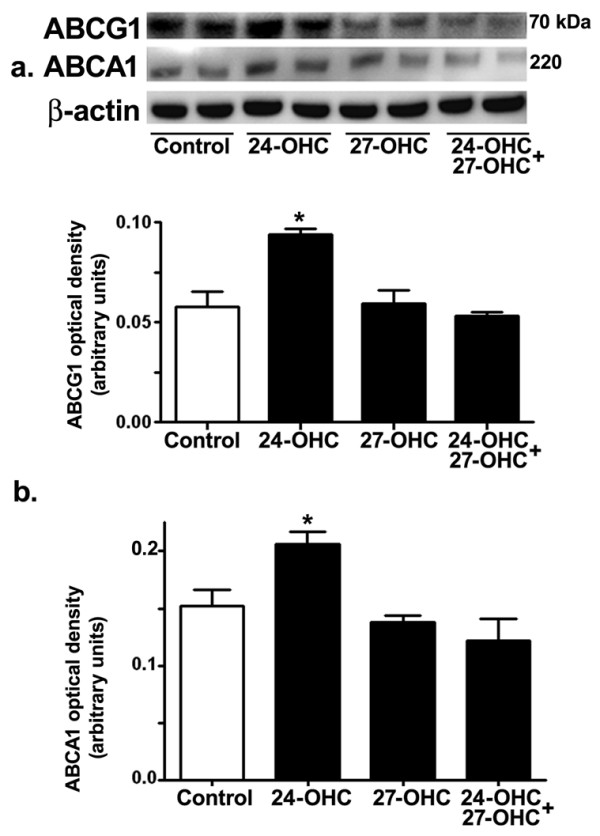
**Treatment with 24-OHC, but not with 27-OHC, increased ABCA1 and ABCG1 levels**. Representative Western blots (a) and densitometric analysis (b) showing increased levels ABCG1 and ABCA1 with 24-OHC treatment. Treatments with 27-OHC or with a mixture of 24-OHC + 27-OHC did not significantly change ABCA1 and ABCG1 levels. *p < 0.05 (One way ANOVA followed by Dunnett's multiple comparison test).

We also determined the effect of treatment with 24-OHC and 27-OHC on ABCG1 and ABCA1, two cholesterol transporters that may be involved in APP processing and Aβ production. Western blot (Fig. 5a) and densitometric (Fig. 5b) analysis show the effect of 24-OHC, 27-OHC, or a mixture of 24-OHC + 27-OHC on levels of ABCG1 and ABCA1. Treatment with 24-OHC significantly increases levels of ABCG1 and ABCA1. Neither 27-OHC nor a mixture of 24-OHC + 27-OHC significantly altered ABCG1 and ABCA1 levels. These results suggest that regulation of ABCG1 and ABCA1 levels with 24-OHC may be involved in the processing of APP to the non-amyloidogenic pathway.

## Discussion

In this study, we demonstrated that 27-OHC favors the amyloidogenic pathway which leads to increased Aβ42 levels. Interestingly, levels of Aβ42 in cells treated with a mixture of 24-OHC + 27-OHC were similar to control levels, suggesting that 24-OHC opposes the increase in Aβ levels triggered by 27-OHC treatment. These effects on Aβ42 may result from differential actions of 24-OHC and 27-OHC on APP levels and processing as 27-OHC, but not 24-OHC, increases levels of APP and BACE1. A previous study has also shown that 24-OHC increases the activity of the non-amyloidogenic pathway [15], and we have recently shown that 27-OHC increases Aβ aggregation in organotypic slices from adult rabbit hippocampus [16].

Abnormalities in cholesterol metabolism in the blood may be important in the pathogenesis of AD (see for review [17]); however, the link between hypercholesterolemia and cerebral levels of Aβ is still obscure. Cholesterol is oxidized to 24-OHC and 27-OHC by the enzymes cholesterol 24-hydroxylase (CYP46A1) and cholesterol 27-hydroxylase (CYP27A1) respectively. While 24-OHC is primarily found in the brain, the amount of 27-OHC is lower in the brain and higher in peripheral circulation [5,6,18]. 27-OHC has the ability to cross the BBB to reach the brain [6]. It may be possible that high blood cholesterol levels are associated with increased turn-over of cholesterol to 27-OHC, a condition that may enhance entry of excess 27-OHC into the brain. High levels of 27-OHC in the brain may therefore be a mechanism by which high cholesterol levels in the blood induce AD-like pathological hallmarks in the brain. Also, reduced levels of 24-OHC in the brain may possibly lead to generation of AD pathology. Indeed, it has been shown that levels of 24-OHC were decreased and levels of 27-OHC were increased in brains from AD subjects as well as in APP Tg mice for AD [5]. Plasma and cerebrospinal fluid levels of 24-OHC were also shown to be higher in early AD and vascular dementia patients in comparison with age-matched controls [19-21]

The cholesterol transporter ABCA1 has been shown to be targeted by oxysterols and linked to Aβ production. ABCA1 suppressed Aβ generation [22] and deletion of ABCA1 led to increased Aβ deposition [23]. A previous study has shown that both 24-OHC and 27-OHC dose dependently increase ABCA1 levels [9]. We demonstrated here an increase in levels of the cholesterol transporters ABCA1 with 24-OHC but not with 27-OHC. This discrepancy may be due to differences in the cell system used. Effects of 24-OHC and 27-OHC on ABCG1, another cholesterol transporter, are less known. One study demonstrated that 24-OHC up-regulates LXR-mediated ABCG1 expression [24]. We demonstrate here that 24-OHC, but not 27-OHC, increases levels of ABCG1. The increase in levels of ABCA1 and ABCG1 is associated with the lower levels of Aβ42 in our study. The effects of 24-OHC on these cholesterol transporters may contribute to the lowering effects by this oxysterol on 27-OHC-induced increase in Aβ42 levels. Conversely, the increase in Aβ42 levels we demonstrated with 27-OHC may be due at least in part to unchanged levels of ABCA1 and ABCG1.

## Conclusion

The present study suggests that 27-OHC increases the accumulation of Aβ by mechanisms that may involve increased processing of APP by BACE1, independently of ABCA1 and ABCG1 expression. Conversely, 24-OHC increases APP processing through the non-amyloidogenic α-secretase pathway, and increases levels of ABCA1 and ABCG1. Further studies are warranted to determine the effect of 24-OHC on Aβ levels in cells overexpressing Aβ or in animal models that exhibit Aβ accumulation. As LXR is the common receptor for 24-OHC and 27-OHC, their differential effects on APP levels and processing may be related to factors other than LXR activation.

## Methods

### Cell culture reagents

Purified 24-OHC was obtained from Biomol International (Plymouth Meeting, PA) and 27-OHC from Medical Isotopes, Inc (Pelham, NH). Stock solutions of 24-OHC and 27-OHC were prepared in ethanol and stored at -70°C. All cell culture reagents were obtained from Invitrogen.

### Cell culture treatment with 24-OHC, 27-OHC, and 24-OHC+27-OHC

Human neuroblastoma SH-SY5Y cells were cultured in 25-cm^2 ^cell culture flasks using Dulbecco's modified Eagle's medium: Ham's F12 with Glutamax (DMEM:F12; 1:1; v/v) and 10% FBS. When the cells reached 80% confluence, they were incubated for 24 h at 37°C in DMEM: F12 with vehicle (ethanol), 24-OHC, 27-OHC, or a mixture (1:1) of 24-OHC and 27-OHC. Cells incubated with vehicles were used as a control.

### Quantification of secreted Aβ levels

Following treatments, the culture medium was collected, supplemented with protease and phosphatase inhibitors cocktail, and centrifuged at 16,000 × *g *for 5 min at 4°C. 100 μl of supernatant was used for Aβ40 and Aβ42 quantification by colorimetric sandwich ELISA (Covance, Denver, PA) according to the manufacturer's protocol. Treatments were performed in triplicate, and the quantity of Aβ in each sample was measured in duplicate and expressed as mean ± standard error for the samples. Aβ40 and Aβ42 levels are expressed in pg/ml.

### Western blot analysis

Control, 24-OHC, 27-OHC, and 24-OHC + 27-OHC treated cells were lysed with a protein extraction reagent (M-PER; Thermo Scientific, Rockford, IL). For sAPPα immunoprecipitation procedure prior to Western blotting, media samples were incubated at 4°C overnight with sAPPα antibody (2B3 clone, IBL-America, Minneapolis, MN) using the Catch and Release Reversible Immunoprecipitation System (Millipore, Billerica, MA). Protein concentrations were determined with the BCA protein assay reagent by standard protocol. Proteins were separated in SDS-PAGE gels, transferred to a polyvinylidene difluoride membrane (Millipore, Bedford, MD) and incubated with antibodies to APP (1:1000, Chemicon International, Temecula, CA), sAPPα (1:100), BACE1 (1:100, Chemicon International, Temecula, CA), ABCG1 (1:100, Novus Biologicals, Littleton, CO), and ABCA1 (1:100, Neuromics, Edina, MN). β-actin was used as gel-loading control. The blots were developed with enhanced chemiluminiscence (Immmun-star HRP chemiluminiscent kit, Biorad, Hercules, CA). The results are quantified by densitometry and represented as total integrated densitometric values.

### Confocal microscopy

The immunostaining with 6E10 for detection of Aβ in control, 24-OHC, 27-OHC, and 24-OHC +27-OHC treated cells was carried out using confocal microscopy. The cells were fixed with paraformaldehyde, blocked with 5% normal goat serum, and reacted overnight at 4°C with 6E10 antibody (1:250, Signet laboratories Inc., Dedham, MA). Cells were then washed and incubated with secondary antibodies conjugated to Alexa fluor-488 (Molecular Probes, Inc., Eugene, OR) for one hour at room temperature in the dark and washed with PBS. The cells were mounted with Vectasheild containing DAPI (Vector laboratories, Inc., Burlingame, CA). The cells were visualized with a Zeiss LSM 510 META confocal system coupled to a Zeiss Axiophot 200 inverted epifluorescence microscope.

### Statistical Analysis

Data was analyzed for statistical significance using analysis of variance (ANOVA) followed by Dunnett's multiple comparison test with GraphPad Prism software 4.01. All values obtained from three different experiments were expressed as mean value ± SEM

## Competing interests

The authors declare that they have no competing interests.

## Authors' contributions

JPRP carried out the ELISA assay and drafted the manuscript, ST performed the Western blot analysis, AH and AT cultured the cells and administered the treatments, ES measured the optical density and helped in the statistical analysis, and OG conceived the study and oversaw the experiments. All authors read and approved the manuscript.
